# Phylo_dCor: distance correlation as a novel metric for phylogenetic profiling

**DOI:** 10.1186/s12859-017-1815-5

**Published:** 2017-09-05

**Authors:** Gabriella Sferra, Federica Fratini, Marta Ponzi, Elisabetta Pizzi

**Affiliations:** Dipartimento di Malattie Infettive, Parassitarie e Immunomediate, Istituto Superiore di Sanità, Viale Regina Elena 299, 00161 Rome, Italy

**Keywords:** Phylogenetic profiling, Distance correlation, Protein-protein interaction

## Abstract

**Background:**

Elaboration of powerful methods to predict functional and/or physical protein-protein interactions from genome sequence is one of the main tasks in the post-genomic era. Phylogenetic profiling allows the prediction of protein-protein interactions at a whole genome level in both Prokaryotes and Eukaryotes. For this reason it is considered one of the most promising methods.

**Results:**

Here, we propose an improvement of phylogenetic profiling that enables handling of large genomic datasets and infer global protein-protein interactions. This method uses the distance correlation as a new measure of phylogenetic profile similarity. We constructed robust reference sets and developed Phylo-dCor, a parallelized version of the algorithm for calculating the distance correlation that makes it applicable to large genomic data. Using *Saccharomyces cerevisiae* and *Escherichia coli* genome datasets, we showed that Phylo-dCor outperforms phylogenetic profiling methods previously described based on the mutual information and Pearson’s correlation as measures of profile similarity.

**Conclusions:**

In this work, we constructed and assessed robust reference sets and propose the distance correlation as a measure for comparing phylogenetic profiles. To make it applicable to large genomic data, we developed Phylo-dCor, a parallelized version of the algorithm for calculating the distance correlation. Two R scripts that can be run on a wide range of machines are available upon request.

**Electronic supplementary material:**

The online version of this article (10.1186/s12859-017-1815-5) contains supplementary material, which is available to authorized users.

## Background

In the last two decades, several computational approaches have been proposed to infer both functional and physical protein-protein interactions (PPIs). These methods includes the identification of gene fusion events [1, 2], conservation of gene neighborhood [3] or phylogenetic profiling [4, 5]. Recently, the increasing number of fully sequenced genomes led to a renewed interest in these approaches. Among them, the phylogenetic profiling is one of the most promising in that it allows to predict protein-protein interactions at a whole genome level, while gene fusion and gene neighborhood are relatively rare events found typically in prokaryotic genomes.

Well implemented methods, based on phylogenetic profiling, have been developed and successfully applied for understanding relationships between proteins and/or to gain insights on the function of uncharacterized proteins [see for example [6–8]. These methods are based on the detection of orthologs either from sequence similarity score or from tree-based algorithms (for a recent implementation see [9]).

In general, phylogenetic profiling is based on the assumption that proteins involved in the same biological pathway or in the same protein complex co-evolve [for a review see [10]. In a first implementation [4], the phylogenetic profile of a protein was defined as a binary vector that describes the occurrence pattern of orthologs in a set of fully sequenced genomes, and the Hamming distance was used to score the similarity between profile pairs. Subsequently, to evaluate different degrees of sequence divergence, phylogenetic profiles were reconstructed using probabilities derived by the expectation values obtained aligning the proteins under study with a genome reference set [5]. Among measures proposed to score the phylogenetic profile similarities [for a review see [11], the Mutual Information (MI) was demonstrated to correlate well in accuracy with genome-wide yeast two-hybrid screens or mass spectrometry interaction assays [5]. Although it was largely adopted as a measure of phylogenetic profile similarity, Simon and Tibshirani recently debated about the lower power of MI in detecting dependency between two variables compared with correlation measures [12].

In this work, we propose the distance correlation (dCor) as a novel metric to score phylogenetic profile similarity. dCor measures any dependence between two variables, ranges between 0 and 1, and it satisfies all requirements of a distance [13, 14].

In order to apply this measure to large genomic data, we developed a novel parallel version of the original algorithm. Furthermore, we adopted a new strategy of genome selection to obtain unbiased and large reference sets of genomes. We applied this methodology to construct phylogenetic profiles of two model organisms, *Escherichia coli* and *Saccharomyces cerevisiae* and confirmed that correlation measures (dCor and Pearson’s correlation) have a more robust predictive performance than the MI. In particular we showed that dCor performs better than Pearson’s correlation (PC) and MI especially in predicting physical protein-protein interactions.

## Implementation

### Phylogenetic profiling

Phylogenetic profiles were obtained as arrays of probability values according to

$$ \boldsymbol{P}=-1/{\boldsymbol{log}}_{10}\left(\boldsymbol{E}\right) $$

For E-values higher than 10^−1^, the probability value is set to 1, as proposed in [5].

Where E are the E-values obtained from the alignments of *S. cerevisiae* and *E. coli* protein sequences against the four reference sets. To do this, we applied the Smith-Watermann alignment algorithm [15]. The FASTA package version 36 was implemented as a stand-alone software on two Work Stations both dual core, the first with 12 CPU and the second with 8 CPU.

### Similarity measures

One of the method usually used to establish similarity between phylogenetic profiles is the mutual information that is calculated according to

$$ MI\left(A,B\right)=H(A)+H(B)-H\left(A,B\right) $$

where *H*(*A*) =  −  ∑ *p*(*a*) ln *p*(*a*) is the summation of the marginal entropies, calculated over the intervals of probability distribution *p*(*a*), of the gene A to occur among the organisms in the reference set. *H*(*A*, *B*) =  −  ∑  ∑ *p*(*a*, *b*) ln *p*(*a*, *b*)represents the summation of the relative entropies of the joint probability distribution *p*(*a*, *b*)of co-occurrence of gene A and B across the set of reference genomes, in the intervals of the probability distribution. The mutual information was calculated by using the *mutualInfo* function available in *bioDist* R package [16] after binning the data into 0.1 intervals.

We calculated dCor according to Szekely and collaborators [13, 14]. The original implementation (available in the energy package of Bioconductor) allows the calculation only between two arrays of data. For this reason, we developed two novel scripts that make possible to perform dCor NxN phylogenetic profile comparison, where N is the number of genes in a given genome. In principle, the method is applicable also to binary phylogenetic profiles.

First, the matrix of the Euclidean distances was obtained calculating the difference between the*k*-th element and the*l*-th element of the phylogenetic profile as

$$ D=\left\lfloor {d}_{kl}\right\rfloor $$

where.

*d*_*kl*_ = |*a*_*k*_ − *a*_*l*_|_*r*_ as the distance between the *r*-th pairs of elements of the profiles.

Second, each distance *d*_*kl*_ of the matrix *D* was then converted into an element *da*_*kl*_ of the matrix of the centered distances *DA*, calculated as

$$ {da}_{kl}={d}_{kl}-{\overline{d}}_k-{\overline{d}}_l+{\overline{d}}_{kl} $$

where.

${\overline{d}}_k=\frac{1}{n}\sum_{k=1}^n{d}_{kl}$ is the average calculated on the rows of the distance matrix;

${\overline{d}}_l=\frac{1}{n}\sum_{l=1}^n{d}_{kl}$is the average calculated on the columns of the distance matrix;

${\overline{d}}_{kl}=\frac{1}{n^2}\sum_{k,l=1}^n{d}_{kl}$is the average calculated on all the elements of the distance matrix;

where *k* = *l* = 1,.…, *n* = 1 ,…, *j*.

The distance correlation between the profiles*A*_*p*_and*A*_*q*_ was calculated as

$$ {dCor}_{pq}=\frac{Cov\left({DA}_p,{DA}_q\right)}{\sqrt{Var\left({DA}_p\right) Var\left({DA}_q\right)}} $$

where *Cov* and *Var*represent the covariance and the variance of the matrices of the centered distances and *p* = *q* = 1 ,  …  , *i*.

Pearson’s correlation was calculated according to

$$ PC=\frac{\sum_{i=1}^n\left({x}_i-\overline{x\ }\right)\ \left({y}_i-\overline{y\ }\right)}{\sqrt{\sum_{i=1}^n\Big({x}_{i\kern0.5em }}-\overline{x}\left)2\sqrt{\sum_{i=1}^n{y}_i-\overline{y}}\right)2} $$

Where *n* is the size of the two arrays x and y, and $\overline{x}$ and $\overline{y}$ are the corresponding means.

#### Gold standards and predictive performance assessment

On the basis of KEGG database [17], we considered proteins belonging to the same metabolic pathway as functional related and hence to be included in the True Positive data set (TP-fun). To derive the True Negative data set (TN-fun), we developed a graph-based algorithm to identify non-interacting proteins. Proteins are included in TN-fun if the length of the shortest path between the metabolic pathways (sub-graphs) they belong was higher or equal to five.

The physically interacting proteins were derived from the STRING database [18]. Protein pairs with evidence about a direct physical interaction were considered as True Positive (TP-phy). True Negative data set (TN-phy) was obtained by applying the graph-based algorithm previously described.

The Area Under the Curve (AUC) was adopted as a measure of the prediction accuracy. The AUC was calculated as the sum of the approximated areas of the trapezoids obtained for each profile similarity score interval, according to the Gini’s formula

$$ AUC=\frac{1}{2}\sum_i\left(\left({X}_i-{X}_{i-1}\right)\left({Y}_i+{Y}_{i+1}\right)\right) $$

where *X*_*i*_ is the false positive rate and *Y*_*i*_ is the true positive rate at the *i*-th interval of profile similarity score. Each interval was set equal to 0.1 of distance correlation or of mutual information and the related rates were calculated. In order to perform the 10-fold cross-validations, each dataset was randomly divided in 10 subsets of equal size and the related AUCs calculated.

The total number of TPs and TNs obtained by dCor, PC and MI calculation in complete data set GS_fun and GS_phy in each reference set is provided in Additional file 1: Table S3.

## Results and discussion

### Reference set construction

It has been shown that the predictive performance of phylogenetic profiling is affected by the size and the genome composition of the reference set [19, 20]. To address this issue, we set up a procedure to construct a reference set that includes a number of genomes sufficiently high to ensure a robust statistics but excludes very similar organisms to avoid redundancy, spanning as much organisms diversity as possible.

To construct genome reference sets, we exploited information in the eggNOG database [17], where 1133 manually selected genomes were collected and classified as “core” (high quality genomes) and “peripheral” (genomes not completely validated) on the basis of genome coverage, status of gene annotation and gene completeness.

The first reference set (RS1) excluded all the strains of the same species classified as “peripheral” genomes. A second reference set (RS2) was generated from RS1 excluding the eukaryotic genomes with a “peripheral” attribute till having 45 eukaryotic genomes in a such way to pass from a ratio 5:1 to a ratio 13:1. To construct the third reference set (RS3), we progressively excluded “peripheral” prokaryotic genomes, in order to obtain the same ratio of RS1 but almost the half size. The last reference set (RS4) was obtained from RS3 on the basis of the Tree of Life derived from the eggNOG database, excluding close phylogenetically related eukaryotic genomes until reaching the same ratio of RS2 (Table 1). In all the four reference set 61 genome from Archea are included. The complete lists of genomes in RS1-RS4 are as Supplemental data (Additional file 2: Table S1).

**Table 1:** Summary of genomes in the reference sets

	Prokaryotes	Eukaryotes	Ratio
Reference set 1 (RS1)	592	120	5:1
Reference set 2 (RS2)	592	45	13:1
Reference set 3 (RS3)	230	45	5:1
Reference set 4 (RS4)	230	18	13:1

In this way, we obtained four reference sets of “high quality” genomes different in size and composition. Using each of the four reference sets, we constructed four phylogenetic profile data sets for *S. cerevisiae* and *E. coli* model genomes and evaluated the effect of the reference set size comparing RS1 vs RS3 and RS2 vs RS4, and composition, comparing RS1 vs RS2 and RS3 vs RS4.

### Phylogenetic profiling

We applied the Smith-Watermann alignment algorithm [15] to align the *S. cerevisiae* and *E. coli* protein sequences against the reference sets. Phylogenetic profiles are constructed as arrays of probability values obtained by the E-values according to

$$ \boldsymbol{P}=-1/{\boldsymbol{log}}_{10}\left(\boldsymbol{E}\right) $$

For E-values higher than 10^−1^, the probability value is set to 1, as proposed in [5]. Phylogenetic profile matrices are available in Supplemental data (Additional file 3: Table S2).

**Fig. 1:**
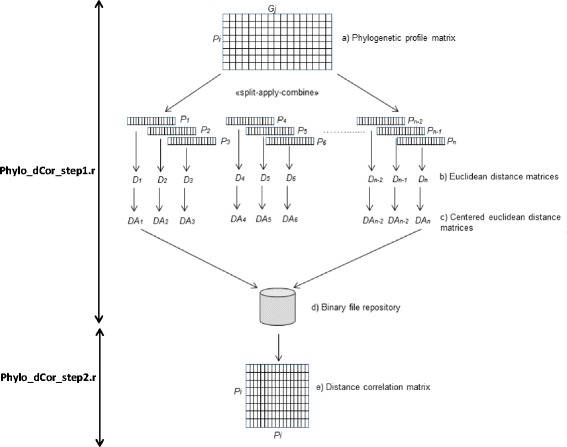
Pipeline of the dCor calculation**.** The phylogenetic profile matrix of Pi proteins constructed using a reference set of size Gj genomes (step a); starting from this data, the D_i_ Euclidean jxj distance matrices (step b) and the DA_i_ centered Euclidean distances (step c) were calculated applying a “split-apply-combine” algorithm; DA_i_ matrices were stored in a repository of binary files (step d), from which they were extracted to proceed with the calculation of the distance correlation matrix (step e)

Comparative analysis of phylogenetic profiling was performed using the dCor [13], the PC and the MI. In order to apply dCor calculation to biological large data sets, we developed a novel algorithm, Phylo_dCor (the strategy is schematically represented in Fig. 1). This proposed implementation strongly reduces the complexity of the original algorithm proposed by Szekelyet al. [13] and hence RAM requirements making it possible to install and run Phylo_dCor on a wide range of machines.

A first script (Phylo_dCor_step1.r) for the R environment was developed to calculate the matrix of centered distances from each phylogenetic profile. First, a phylogenetic profile matrix Pi x Gj was constructed where Pi are the probability values calculated for each hit found in the Gj genomes of the reference set (step a). Then, we adopted a “split-apply-combine” strategy using the *plyr* R package [21]. This allowed us to parallelize the most “time-consuming” steps subdividing the Pi xGj matrix into N sub-matrices and hence the calculations of the Euclidean distance matrices (step b) and of the Euclidean centered distance matrices (step c). The resulting matrices of centered distances were stored in a repository of binary files (.rds) (step d). A second R code (Phylo_dCor_step2.r) was developed to perform the calculation of the distance correlation (step e).

To evaluate the performance of the method, a ten-fold cross-validation procedure was carried out on two different sets of gold-standards. The first set was derived from the metabolic pathways in KEGG database [22], and includes as TPs pairs of functionally related proteins (GS_fun), the second set was obtained from the STRING database [18], to assess the performance in predicting physical protein-protein interactions (GS_phy). The predictive performance was estimated by calculating the Area Under the ROC Curve (AUC) values for each of the 10 randomly selected independent subsets.

The analysis was performed on all proteins deduced from the two model genomes, including paralogs and possible horizontal gene transfers. Being them considered in all the three assessments, the comparative predictive performance of dCor, PC and MI was not affected. Moreover, possible false positives can be evaluated and eventually filtered away in a second step.

**Fig. 2:**
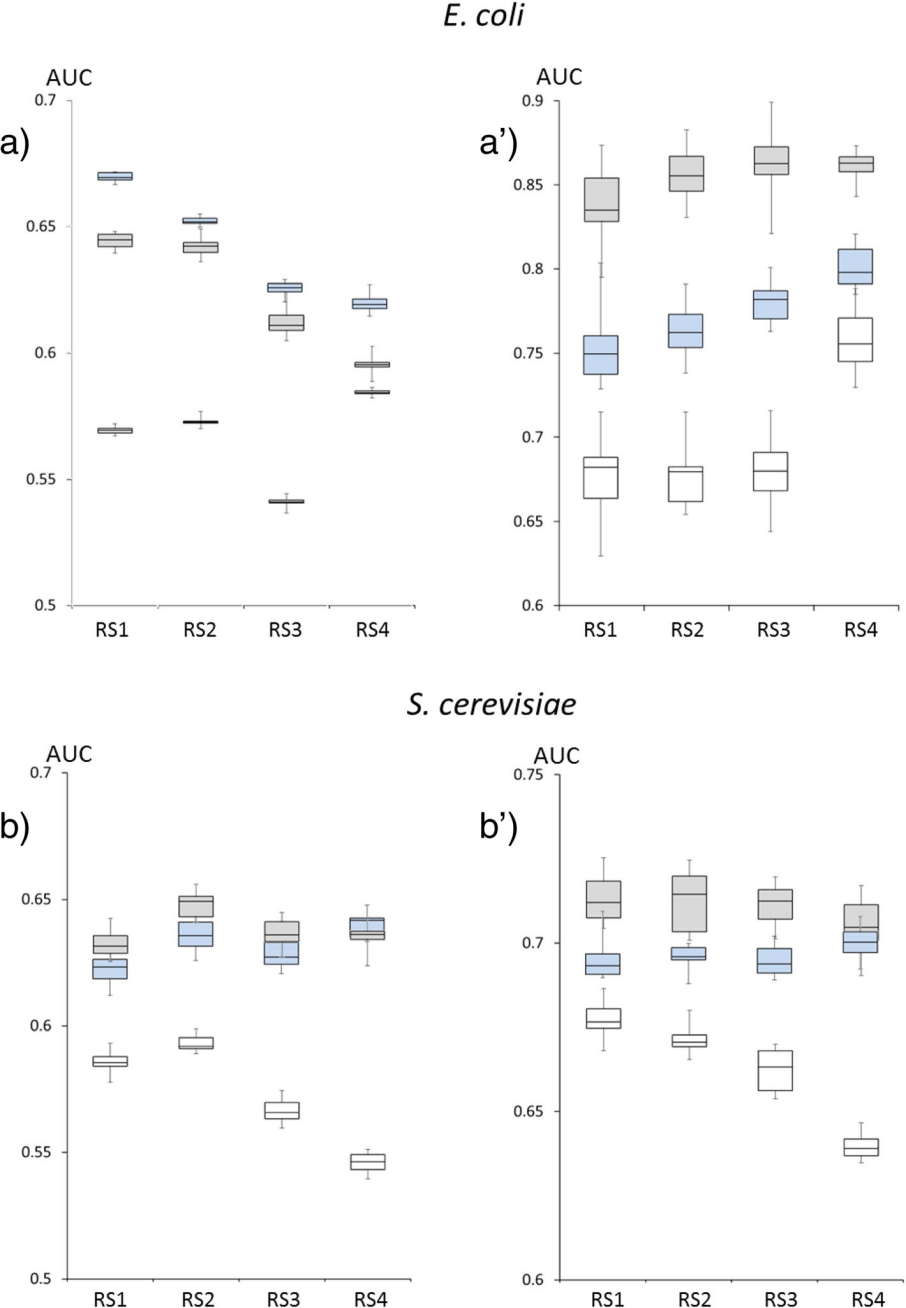
Benchmarking of Phylo-dCor application**.** Results of the ten-fold cross-validation procedure to assess predictive performances of dCor (grey box plots), PC (ligth blue box plots) and MI (empty box plots). Results obtained using GS_fun benchmark are shown in panels **a** and **b**, while in panels **a’** and **b’** are reported results obtained using GS_phy

In Fig. 2 results regarding the assessment on GS_fun are shown in panels a and b, while results obtained using GS_phy are reported in panel a’ and b’. In all cases but one, the predictive performance of the phylogenetic profiling using dCor (grey box-plot) outperforms the one obtained using MI (empty box-plot) and PC (ligth blue box-plot). We confirmed that both size and composition of the reference set affect phylogenetic profiling. However, the use of dCor and PC to compare phylogenetic profiles strongly reduces this effect, especially in the case of the eukaryotic genomes. In general, it seems that physical interactions (Fig. 2, panels a’ and b’) are predicted better than functional relationships. This could be due to a higher robustness of the gold standards GS-phy than GS-fun, in that physical interactions are experimentally validated. PC outperforms dCor in the case of the GS-Fun gold standard in *E. coli*, furthermore in this case the effect of the size and/or genome composition of the reference sets affects also the predictive performance of correlation measures.

Collectively, our results indicate that the proposed application is robust, and significantly improves the performance of PPI prediction. It can efficiently handle large genomic data sets and does not require high calculation capacity.

## Conclusions

The increasing number of fully sequenced genomes led to a renewed interest in the elaboration of powerful methods to predict both functional and physical protein-protein interactions. In this framework, we propose a novel phylogenetic profiling procedure using distance correlation as a similarity measure of phylogenetic profiles. To make it applicable to large genomic data, we developed Phylo-dCor, a parallelized version of the original algorithm for calculating the distance correlation. Two R scripts that can be run on a wide range of machines will be made available on request. Furthermore, we adopted a new strategy of genome selection to obtain unbiased and large reference sets of genomes. In two model genomes: *E. coli* and *S. cerevisiae* we showed that the distance correlation outperforms phylogenetic profiling methods previously described.

## Additional files

Additional file 1: Table S3. Table of TPs and TNs. The number of True Positives and True Negatives obtained by dCor, MI and PC calculation for each reference set and each gold standard (GS-fun and GS-phy) for *E. coli* and *S.cerevisiae. (XLSX 23 kb)*

Additional file 2: Table S1. List of reference set genomes. The complete lists of genomes utilized for construction of reference sets RS1-RS4. (XLSX 85 kb)

Additional file 3: Table S2. Phylogenetic profile matrices. The phylogenetic profiles derived for *E. coli* and *S. cerevisiae* using the reference set RS1. (XLSX 68277 kb)

## Abbreviations

dCor: distance correlation
MI: Mutual Information
PC: Pearson’s correlation
RS: Reference Set
TN: True Negative
TP: True Positive
